# Balanced forced-diuresis compared to control as a reno-protective approach in cardiac surgery: secondary outcome of a randomized controlled trial, assessment of neutrophil gelatinase-associated lipocalin levels

**DOI:** 10.1186/s13019-021-01620-w

**Published:** 2021-08-24

**Authors:** Heyman Luckraz, Ramesh Giri, Benjamin Wrigley, Kumaresan Nagarajan, Eshan Senanayake, Emma Sharman, Lawrence Beare, Alan Nevill

**Affiliations:** 1 Heart-Centre, American Hospital, PO Box 5566, Dubai, UAE; 2 Heart and Lung Centre, Wolverhampton, WV10 0QP UK; 3 University Hospitals of North Midlands, Stoke-on-Trent, ST4 6QG UK; 4 Faculty of Education, Health and Wellbeing, University of Wolverhampton, Wolverhampton, WS1 3BD UK

**Keywords:** Acute kidney injury, Cardiac surgery, RenalGuard® system, Neutrophil gelatinase-associated lipocalin, Balanced forced-diuresis

## Abstract

**Background:**

Neutrophil gelatinase-associated lipocalin (NGAL) is a recognised biomarker for acute kidney injury (AKI).This study investigated the impact of balanced forced-diuresis using RenalGuard® system (RG), in reducing acute kidney injury (AKI) rates and the associated NGAL levels (6-h post-CPB plasma level) post adult cardiac surgery with cardiopulmonary bypass (CPB).

**Methods:**

Patients included in the study were at high-risk for AKI post cardiac surgery, namely history of diabetes and/or anaemia, e-GFR 20–60 ml/min/1.73 m^2^, Logistic EuroScore > 5, anticipated CPB time > 120 min. Patients were randomized to either RG (n = 110) or managed as per current practice (control = 110). RIFLE-defined AKI rate (based on serum creatinine level increase) within first 3 days of surgery and 6-h post CPB NGAL levels were the primary and secondary end-points.

**Results:**

Pre and intra-operative characteristics between the two groups were similar (p > 0.05) including the pre-op NGAL levels, the oxygen delivery (ecDO_2_i) and the carbon dioxide production (ecVCO_2_i) during CPB. Patients in the RG group had a significantly lower post-operative RIFLE-defined AKI rate compared to control (10% (11/110) v/s 20.9% (23/110), p = 0.03). Overall, median 6-h post CPB NGAL levels in patients with AKI were significantly higher than those who did not develop AKI (211 vs 150 ng/ml, p < 0.001). Patients managed by balanced forced-diuresis had lower post-operative NGAL levels (146 vs 178 ng/ml, p = 0.09). Using previously reported NGAL cut-off level for AKI (142 ng/ml), binary logistic regression analysis confirmed a beneficial effect of the RG system, with an increased risk of AKI of 2.2 times in the control group (OR 2.2, 95% CI 1.14–4.27, p = 0.02).

**Conclusions:**

Overall, the 6-h post-CPB plasma NGAL levels were significantly higher in patients who developed AKI. Patients managed with the novel approach of balanced forced-diuresis, provided by the RenalGuard® system, had a lower AKI rate and lower NGAL levels indicating a lesser degree of renal tissue injury.

*Trial registration* ClinicalTrials.gov website, NCT02974946, https://clinicaltrials.gov/ct2/show/NCT02974946.

## Background

Neutrophil gelatinase-associated lipocalin (NGAL) was first described in 2003 as a urinary marker of ischaemia-related renal injury [1]. It is as a 25-kD protein covalently bound to gelatinase from human neutrophils [2], expressed in stimulated epithelia of kidney (ischemia–reperfusion injury), stomach and colon (inflammation or neoplasia) and in the lungs (chest infections, asthma, emphysema [3]. It promotes proliferation in the kidney cell tubules via cell modulation, inflammation and neoplastic transformation as well as reducing apoptosis [3]. After an ischaemic renal insult, NGAL is expressed by epithelial cells of the thick ascending limb and collecting ducts of the nephrons.

Acute kidney injury (AKI) is well documented after cardiac surgery [4]. It is possibly related to the effect of vasoconstriction and haemodilution when patients are on the cardiopulmonary bypass machine (CPB). Although several strategies have been applied to reduce AKI [5], the latter still accounts for significant morbidity and mortality [6].

Clinical use of NGAL as an early bio-marker of AKI has been investigated especially in the paediatric population undergoing cardiac surgery where the cardiopulmonary bypass machine is used [7]. NGAL levels can be measured in both urine and plasma [8] and can potentially provide early recognition of AKI so that preventive measures could be implemented. One such novel preventive approach can be provided by the RenalGuard® system (RG) (RenalGuard Solutions Inc., Milford, USA). This device uses the principle of forced-diuresis and the operator can set the desired fluid-replacement level to even, positive or negative balance. The various aspects of the device have been described in details previously [9]. Its use in adult cardiac surgery has also been reported and the clinical benefit confirmed [10]. In brief, the RG system is a closed-loop system of urine collection via a Foley’s catheter and an intravenous fluid administration-set interfaced through a monitoring console to adjust for even, positive or negative fluid balance.

This study reports on the impact of forced-diuresis with furosemide and instantaneously-matched even-balanced intravenous rehydration using the RenalGuard® system on its secondary outcomes of the changes in the NGAL levels (6-h post CPB) as well as the clinical outcomes after cardiac surgery.

## Materials and methods

### Aims and objectives

The primary end-point of the study was recently reported and published [10]. It was the RIFLE-defined AKI rate (criteria definition—50% increase in pre-op “baseline” serum creatinine within first 3 days of surgery) comparing patients managed with the RG system to controls. Baseline serum creatinine level was defined as latest serum creatinine level available prior to surgery. Secondary objectives included changes in plasma NGAL levels (Bioporto Diagnostics, Hellerup, Denmark) at 6-h post-CPB as well as pre-CPB NGAL levels and post-operative complications.

### Ethical approval

This study (KIDNEY) was reviewed and approved by the Institutional Research Committee (16CARD13) prior to seeking Ethical committee (16/NI/0246, 2^nd^ December 2016) approval and was registered on ClinicalTrials.gov website (NCT02974946). The study was also supported by the National Institute of Healthcare Research, Clinical Research Network, United Kingdom (NIHR ID: 32769). All recruited patients gave written informed consent to partake in the study. Trial patients were treated according to the Declaration of Helsinki 2013.

### Inclusion criteria

Patients with one (or more) of the following risk factors for AKI were recruited for this study: (1) diabetics (insulin or non-insulin dependent diabetes mellitus), (2) haemoglobin level of 12.5 g/dl or below, (3) Logistic Euroscore of 5 or above, (4) estimated glomerular filtration rate (eGFR) of 20–60 mL/min/1.73 m^2^ and (5) cardiac procedures when CPB-time was likely to exceed 120 min. Recruited patients underwent one of the following procedures: isolated coronary artery bypass grafting (CABG), isolated valve surgery (aortic valve replacement—AVR, mitral valve replacement—MVR, mitral valve repair—MV repair) or combined procedures (CABG, AVR, MV repair, tricuspid valve repair, MVR, Cox-Maze IV atrial fibrillation ablation, ascending aorta replacement, aortic root surgery, left ventricular aneurysmectomy and myxoma surgery in various combinations).

### Intervention

Consented patients were randomized using sealed opaque envelopes system generated by a research independent person in the research and development department. Management of patients randomised to the RenalGuard® system has been previously described [10] and included instantaneously-matched balanced forced-diuresis with furosemide and intravenous rehydration with Hartmann’s solution. Patients in the control group were managed as per current medical practice without forced-diuresis with intravenous furosemide in the OR. Otherwise, the management of the patients was similar including the anaesthetic technique and cardiopulmonary bypass run including the need for inotropic support to maintain a MAP (mean arterial pressure) > 65 mmHg. CPB-flow was maintained at cardiac index of 2.4 l/min/m^2^. Oxygen delivery (ecDO_2_i) and CO_2_ production (ecVCO_2_i) during the CPB were monitored using the System M4 (Spectrum Medical, Gloucester, UK). Post-operatively both groups of patients were transferred to CICU.

### NGAL test

The NGAL levels were assessed from plasma (ethylene diamine tetra-acetic acid—EDTA—tube) collected prior to the start of cardiac surgery and at 6-h post cessation of CPB while the patient was recovering on the CICU. Specimens were centrifuged at 2380 g for 10 min at 4 °C on the day of receipt, to pellet cellular material and the supernatants aliquoted and stored at − 80 °C until analysed. The NGAL test was a quantitative particle-enhanced turbimetric immune-assay (PETIA) using the BioPorto Kit (BioPorto Diagnostics A/S, Gentofte, Denmark) and the Abbott Architect C16000 analyser (Abbott Diagnostics, Chicago, USA).

### Power calculations and statistical analyses

Based on the data from the department’s cardiac surgery database and previous publications [6], 110 patients per group were deemed adequate to achieve the primary end-point (RIFLE defined AKI rate) with a power of 0.8 and an alpha of 0.05.

Categorical data are expressed as percentages and differences between the two groups assessed using the *X*^2^ test of independence. Continuous variables are expressed as mean (standard deviation—SD) or median (minimum, maximum) for normally and skewed distributed data respectively. Group comparison was carried out using two-tailed t-test or Mann–Whitney U test accordingly. Adopting a previously reported NGAL cut-off level (142 ng/ml) for AKI, binary logistic regression analysis was used to assess the beneficial effect of the RG system. The factors included in the model were age, gender, ethnic origin, history of diabetes, left ventricular ejection fraction, pre-operative creatinine level, pre-operative NGAL level, pre-op eGFR level, Log EuroScore, STS Score, study group (RG vs. control), CVP, MAP and lactate levels in OR prior to start of surgery, CPB duration, procedure performed, urine volume produced in OR, CVP, MAP and lactate levels on admission to CICU and blood transfusion. The tests were considered significant at p < 0.05. SPSS version 26.0 (IBM SPSS statistics 26) was used for statistical analyses.

Two patients who were randomized to RG group could not be catheterized per urethrally at the start of the operation and had a supra-pubic catheter inserted at the end of the procedure and hence were not connected to the RG system. Additionally, two patients in the RG group and one patient from the control group had their surgery cancelled and therefore did not have post-operative data. All patients’ data were analysed on an intention- to-treat basis.

**Fig. 1:**
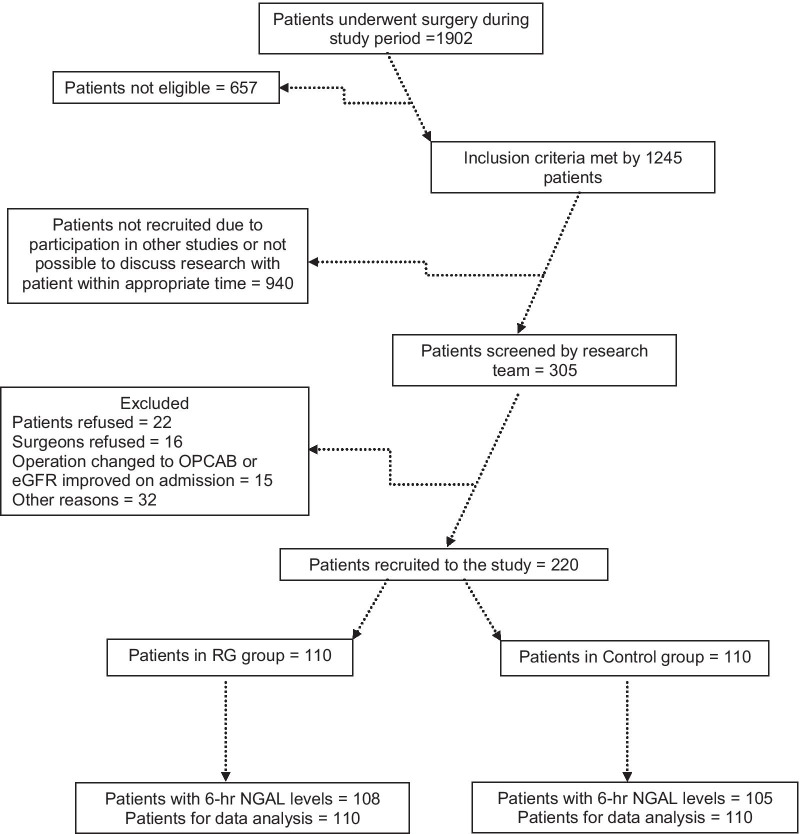
CONSORT flow diagram for recruitment

Patients were recruited from 1st March 2017 to 4th September 2019 as shown in the Consolidated Standards of Reporting Trials (CONSORT) flow diagram (Fig. 1).

## Results

Two hundred and twenty patients undergoing cardiac surgery were recruited to the study (110 patients per group). The patients’ pre and intra-operative characteristics are detailed in Table 1 and were not significantly different between the two groups.

**Table 1:** Pre, peri and post-operative characteristics of patients in the two groups

	RG group (n = 110)	Control group (n = 110)	p value
Pre-op creatinine^a^, micromol/L	99.0 (28.2)	98.3 (34.7)	0.29
Pre-op eGFR^a^, ml/min/1.73m^2^	66.5 (17.2)	68.2 (20.3)	0.47
Pre-op Haemoglobin^a^, g/L	135 (16)	134 (18)	0.72
Logistic EuroScore^b^	3.8 (0.9, 67.4)	3.2 (0.9, 32.5)	0.3
Age^a^, years	67.8 (9.3)	67.0 (9.2)	0.33
Pre-op NGAL level^b^, (ng/ml)	83 (19, 202)	86(15,459)	0.64
Male, (n, %)	87, 79%	84, 76%	0.63
Non-diabetics, (n, %)	30, 27%	30, 27%	0.66
Impaired LVEF, (n, %)	51, 46%	38, 34%	0.14
Urgent surgery, (n, %)	34, 31%	24, 22%	0.13
Combined operations, (n, %)	40,37%	31, 28%	0.38
ecDO_2_i^a^, ml/min/m^2^	300 (52)	300 (52)	0.73
ecVCO_2_^a^, ml/min/m^2^	70 (24)	71 (21)	0.92
XCT time^a^, mins	84 (40)	79 (42)	0.25
Serum lactate during surgery^b^, mmol/L	1.97 (0.78,5.00)	1.80 (0.87,4.98)	< 0.01
Serum lactate on CICU^b^, mmol/L	1.8 (0.60, 6.80)	1.7 (0.6, 8.70)	0.14
CICU stay^b^, hours	27 (16, 410)	28 (8, 486)	0.92
Atrial Fibrillation, (n/total, %)	12/108, (11%)	10/108, (9%)	0.62
Blood transfusion, (n/total, %)	29/108, (27%)	21/109, (19%)	0.16
Infection, (n/total,%)	10/108, (9%)	8/108, (7%)	0.59
In-hospital survival, (n/total, %)	109/110 (99%)	108/110 (98%)	0.56

Table showing the patients’ characteristics for the two groups along with the intra-operative data and post-operative complications rates

*e-GFR* estimated glomerular filtration rate, *NGAL* neutrophil gelatinase-associated lipocalin, *LVEF* left ventricular ejection fraction, *ecDO2i* oxygen delivery, *ecVCO2i* CO2 production during the CPB, *XCT* cross clamp time, *CICU* cardiac intensive care unit

^a^Denotes mean (SD)

^b^Denotes median (minimum, maximum)

The average oxygen delivery (ecDO_2_i) and CO_2_ production (ecVCO_2_i) during the CPB were maintained at similar levels for the two groups, Table 1. Likewise the MAP and the central venous pressure (CVP) were not significantly different during CPB being 57.5 (8.9) vs 57.5 (9.4) mmHg and 3 (4.9) vs 3 (4.6) cmH_2_O respectively. Although the median lactate levels during surgery were statistically different between the two groups, these levels were not clinically significantly different, being around 2 mmol/L (Table 1).

Post-operative AKI rates (as per RIFLE definition) were significantly lower in RG group as compared to control (10% (11/110) v/s 20.9% (23/110), p = 0.025) and has, previously, been reported in details [10].

The secondary end-points confirmed that mean volumes of urine produced during surgery were significantly higher in RG group (2366 ± 877 ml v/s 765 ± 549 ml, p < 0.001). Likewise at 6-h post- CICU admission, patient in the RG group produced a higher volume of urine (1911 ± 904 ml v/s 911 ± 407 ml, p < 0.001). Moreover, the median 6-h post-CPB NGAL levels were significantly higher in the patients who developed AKI (211 vs 150 ng/ml, p < 0.001). Generally, patients managed with balanced forced-diuresis had lower post-op median NGAL levels (146 vs 178 ng/ml, p = 0.09).

When previously reported NGAL cut-off level (142 ng/ml) for AKI was used [11], binary logistic regression analysis confirmed a beneficial effect of the use of the RG system, with an increased risk of AKI of 2.2 times in the control group (OR 2.2, 95% CI 1.14–4.27, p = 0.02). Higher levels of 6-h post-op NGAL were also associated with pre-operative NGAL level, Log EuroScore and being in the control group as noted above. There was no significant direct correlation between the serum creatinine levels and the plasma NGAL levels pre-operatively or post-operatively (Table 2).

**Table 2:** Correlation between serum creatinine level and plasma NGAL level

Pearson correlation	RG group	Control group
Pre-op creatinine and pre-op NGAL	0.476	0.43
RIFLE creatinine and 6-h NGAL (post-op)	0.518	0.587
Creatinine and NGAL changes^a^	0.405	0.163

^a^Denotes the ratio of the post-op change of the level from baseline

During the post-operative recovery phase, there was no significant difference in incidence of atrial fibrillation rate, blood transfusions and infections (Table 1) between the 2 groups. One patient in RG group and two patients in control group died prior to hospital discharge with the cause of death not related to the use of device.

## Discussion

This study confirmed that, in patients at-risk of AKI post cardiac surgery, balanced forced-diuresis as provided by the RenalGuard® system reduces the AKI rate and the associated 6-h post-CPB NGAL levels.

NGAL has been likened to the troponin-equivalent of the kidneys for AKI detection. It is a reflection of tissue damage within the renal system [1]. A rise in the NGAL levels within the first few hours of the insult has provided the possibility of an early diagnosis of AKI post-CPB. Some researchers have even advocated the use of both the pre-operative and post-operative NGAL levels as a means to stratify clinical management strategies [12]. Both urinary and plasma NGAL levels have been evaluated [8, 13] and currently, plasma NGAL is emerging as the most reliable option [14]. In the current study, patients who developed AKI had significantly higher 6 h-post CPB NGAL levels. The use of the RG system was associated with lower NGAL levels post-op but it did not reach statistical significance. This could be a reflection of the fact the study was powered to assess AKI rates rather than the 6 h-post CPB NGAL levels. A larger size study would be needed to confirm these NGAL-level findings. Based on the current data available from this study, a sample size of 200 patients per group would be needed to test this null hypothesis (assessment of a difference in the 6-h post-CPB NGAL levels between RG and control) with a power of 0.8 and an alpha of 0.05.

It is important to ascertain that organ perfusion is optimized during the CPB run. There is good evidence to suggest that AKI during CPB is related to significant impairment of renal oxygenation due to renal vasoconstriction and haemodilution [15]. Traditionally, MAP, CVP, CPB flow-rates and overall blood oxygen saturation had been used as indicators of tissue perfusion but more recently measurement of oxygen delivery has provided an improved level of tissue perfusion assessment. Ranucci et al. have reported that oxygen delivery levels below 272 ml/min/m^2^ was the strongest predictor of AKI [16]. Similar findings were reported by Rasmussen et al. who reported at least a 2.5 times increase in AKI rate when ecDO_2_i was less than 272 ml/min/m^2^ [17]. It has also been reported that ecVCO_2_i level exceeding 60 ml/min/m^2^ was associated with hyperlactataemia indicating poor tissue perfusion [18]. In this study, not only the MAP, CVP and CPB flow-rates but also the ecDO_2_i and ecVCO_2_i were maintained at appropriate and similar levels for both groups ensuring adequate organ perfusion (Table 1). During this study, the ecDO_2_i/ecVCO_2_i ratio was maintained above 5, ensuring effective organ perfusion during the CPB. Furthermore, the median lactate levels during CPB and within the first 6 h on the CICU were less 2 mmol/L for both groups (Table 1).

The mechanisms by which this balanced forced-diuresis, in the RG group, would protect the kidneys is not entirely clear but could be related to the fact that furosemide prevents renal hypoxia at the level of loop of Henle by inhibiting the sodium potassium chloride co-transporter (NKCC2) channels present on the apical membrane of the thick ascending limb (TAL) [19] whilst the forced-diuresis prohibits the build-up of casts within the renal tubules which is well recognized in acute tubular necrosis. The patients in the RG group produced three times as much urine during surgery, and twice as much within the first 6 h on CICU, as compared to controls. This constant high-volume diuresis could potentially prevent the clogging of the renal tubules but could lead to intra-vascular dehydration of the patient. Thus, it is imperative to maintain real-time instantaneous fluid replacement as provided by the RG system.

As expected, there was no significant correlation between the plasma NGAL levels and the serum creatinine levels in the two groups. This is hardly surprising as the serum concentration of creatinine represents a balance between its production (related to muscle mass) and the glomerular filtration rate (GFR) while the plasma NGAL is reflective of ischaemic renal insult leading to NGAL being expressed by epithelial cells of the thick ascending limb and collecting ducts of the nephrons. Serum creatinine level is an insensitive, non-specific and delayed marker of acute kidney injury and the level is influenced by several factors such as muscle mass, muscle damage, nutritional status and body surface area as well as medications which affect the glomerular filtration rate [20]. Plasma NGAL however is a more sensitive marker of acute kidney injury and may become in the near future the new gold standard for assessment of AKI.

Finally, despite the large volume diuresis with the corresponding volume fluid replacement that the RG group had as compared to controls, there was no significant difference in the requirements for blood transfusion between the two groups. Similarly, the rates of atrial fibrillation as well as infections were similar in both groups. This confirms that the use of balanced forced-diuresis is safe in this group of surgical patients.

This study now provides additional information (secondary outcome) regarding the possible mechanisms of the renal-protective effect of a novel therapy using balanced forced-diuresis, provided by the RenalGuard® system, by reducing ischaemic kidney injury as reflected in the lower 6 h-post CPB NGAL levels. It has already reported reduced AKI rates when the RenalGuard® system was used [10].

### Limitations

This is a single-centre study and is now reporting on the secondary outcomes. The study was powered for the primary end-point only. Additionally, it was not possible to blind the attending clinical personnel to the study.

## Conclusion

Overall, the 6-h post-CPB plasma NGAL levels were significantly higher in patients who developed AKI. RenalGuard® system reduces the 6 h-post CPB NGAL levels by using the principle of balanced forced-diuresis. Patients managed with this novel approach (balanced forced-diuresis) had a lower AKI rate and lower NGAL levels indicating a lesser degree of renal tissue injury.

## Data Availability

The datasets used and/or analysed during the current study are available from the Research and Development Department, Heart and Lung Centre, Wolverhampton, on reasonable request.

## Abbreviations

AKI: Acute kidney injury
AVR: Aortic valve replacement
CABG: Isolated coronary artery bypass grafting
CICU: Cardiac intensive care unit
CONSORT: Consolidated Standards of Reporting Trials
CPB: Cardiopulmonary-bypass machine
CVP: Central venous pressure
ecDO_2_i: Indexed extra-corporeal circuit oxygen delivery
ecVCO_2_i: Indexed extra-corporeal circuit carbon dioxide production
EDTA: Ethylene diamine tetra-acetic acid
e-GFR: Estimated-glomerular filtration rate
i.v: Intravenous
KIDNEY study: **K**idney protect**i**on using the RenalGuar**d**® system i**n** cardiac surg**e**r**y**
MAP: Mean arterial pressure
MVR: Mitral valve replacement
MV: Repair mitral valve repair
NKCC2: Sodium potassium chloride co-transporter
NGAL: Neutrophil gelatinase-associated lipocalin
OR: Operating room
PETIA: Particle-enhanced turbimetric immune-assay
RG: RenalGuard® system
RIFLE: Risk, injury, failure, loss of kidney function, end-stage renal disease
SD: Standard deviation
TAL: Thick ascending limb
